# Supplementary material for: Causal Relationship between Aging and Anorexia Nervosa: A White-Matter-Microstructure-Mediated Mendelian Randomization Analysis
Source: Biomedicines. 2024 Aug 16;12(8):1874. doi: 10.3390/biomedicines12081874 (PMC11351342; doi:10.3390/biomedicines12081874)
Supplement: Supplementary file 1 [file biomedicines-12-01874-s001.zip › Supplementary Figure/Figure S4/Singe SNP plot of MR analysis for TL to ubm-b-1945.pdf]

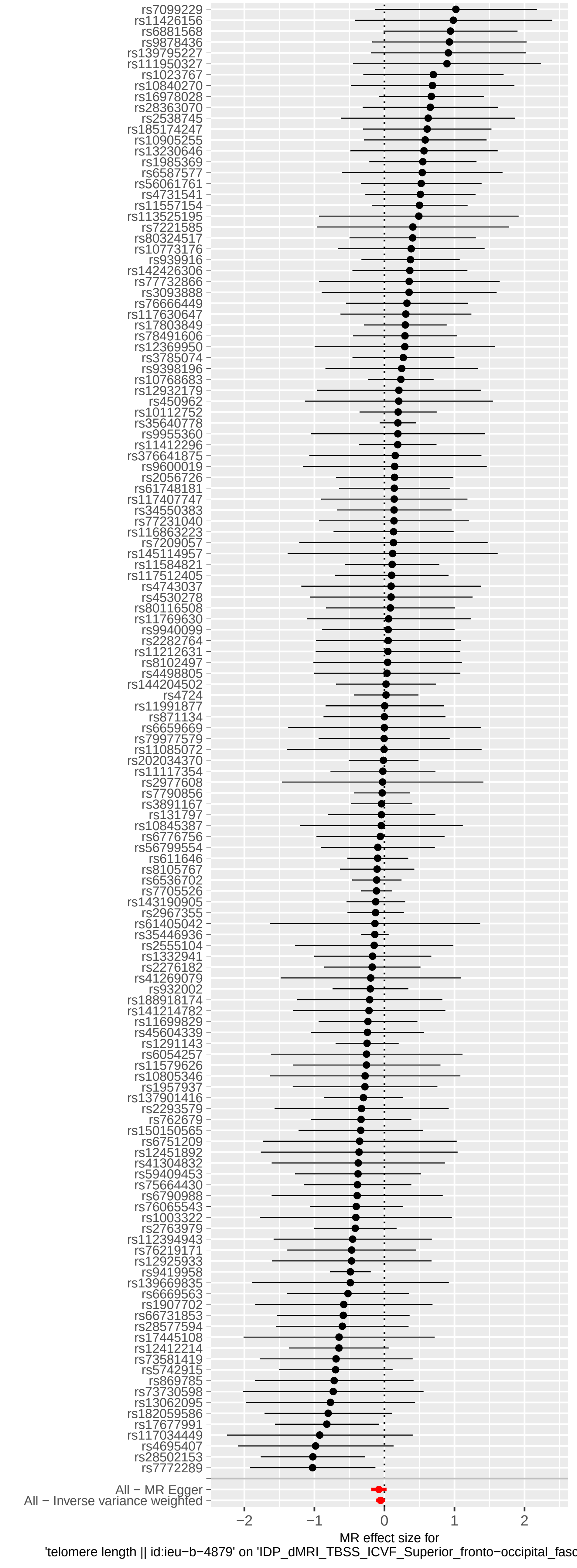
rs7099229
rs11426156
rs6881568
rs9878436
rs139795227
rs111950327
rs1023767
rs10840270
rs16978028
rs28363070
rs2538745
rs185174247
rs10905255
rs13230646
rs1985369
rs6587577
rs56061761
rs4731541
rs11557154
rs113525195
rs7221585
rs80324517
rs10773176
rs939916
rs142426306
rs77732866
rs3093888
rs76666449
rs117630647
rs17803849
rs78491606
rs12369950
rs3785074
rs9398196
rs10768683
rs12932179
rs450962
rs10112752
rs35640778
rs9955360
rs11412296
rs376641875
rs9600019
rs2056726
rs61748181
rs117407747
rs34550383
rs77231040
rs116863223
rs7209057
rs145114957
rs11584821
rs117512405
rs4743037
rs4530278
rs80116508
rs11769630
rs9940099
rs2282764
rs11212631
rs8102497
rs4498805
rs144204502
rs4724
rs11991877
rs871134
rs6659669
rs79977579
rs11085072
rs202034370
rs11117354
rs2977608
rs7790856
rs3891167
rs131797
rs10845387
rs6776756
rs56799554
rs611646
rs8105767
rs6536702
rs7705526
rs143190905
rs2967355
rs61405042
rs35446936
rs2555104
rs1332941
rs2276182
rs41269079
rs932002
rs188918174
rs141214782
rs11699829
rs45604339
rs1291143
rs6054257
rs11579626
rs10805346
rs1957937
rs137901416
rs2293579
rs762679
rs150150565
rs6751209
rs12451892
rs41304832
rs59409453
rs75664430
rs6790988
rs76065543
rs1003322
rs2763979
rs112394943
rs76219171
rs12925933
rs9419958
rs139669835
rs6669563
rs1907702
rs66731853
rs28577594
rs17445108
rs12412214
rs73581419
rs5742915
rs869785
rs73730598
rs13062095
rs182059586
rs17677991
rs117034449
rs4695407
rs28502153
rs7772289
All − MR Egger
All − Inverse variance weighted
−2
−1
0
1
2
MR effect size for
'telomere length || id:ieu−b−4879' on 'IDP_dMRI_TBSS_ICVF_Superior_fronto−occipital_fasc
